# Phylogenetic trees, conserved motifs and predicted subcellular localization for transcription factor families in pearl millet

**DOI:** 10.1186/s13104-023-06305-2

**Published:** 2023-03-20

**Authors:** Yingwei Qu, Ambika Dudhate, Harshraj Subhash Shinde, Tetsuo Takano, Daisuke Tsugama

**Affiliations:** 1 Asian Research Center for Bioresource and Environmental Sciences (ARC-BRES), Graduate School of Agricultural and Life Sciences, The University of Tokyo, 1-1-1 Midori-cho, Nishi-tokyo-shi, 188-0002 Tokyo, Japan; 2 Stowers Institute for Medical Research, 1000 East 50th Street, 64110 Kansas City, issouri USA; 3 University of Kentucky, 40506 Lexington, Kentucky, USA

**Keywords:** Pearl millet, Transcription factor, Phylogenetic analysis, Protein family, Subcellular localization, Protein domain, Motif

## Abstract

**Objectives:**

Pearl millet (*Pennisetum glaucum*) is a cereal crop that is tolerant to a high temperature, a drought and a nutrient-poor condition. Characterizing pearl millet proteins can help to improve productivity of pearl millet and other crops. Transcription factors in general are proteins that regulate transcription of their target genes and thereby regulate diverse processes. Some transcription factor families in pearl millet were characterized in previous studies, but most of them are not. The objective of the data presented was to characterize amino acid sequences for most transcription factors in pearl millet.

**Data description:**

Sequences of 2395 pearl millet proteins that have transcription factor-associated domains were extracted. Subcellular and suborganellar localization of these proteins was predicted by MULocDeep. Conserved domains in these sequences were confirmed by CD-Search. These proteins were classified into 85 families on the basis of those conserved domains. A phylogenetic tree including pearl millet proteins and their counterparts in *Arabidopsis thaliana* and rice was constructed for each of these families. Sequence motifs were identified by MEME for each of these families.

## Objective

Pearl millet (*Pennisetum glaucum*) is a staple cereal crop that is tolerant to a high temperature, a drought and a poor-nutrient condition and that is produced in semi-arid regions [1]. Characterization of pearl millet genes can help to better understand pearl millet stress tolerance and to improve productivity of pearl millet and other crops. The whole genome sequence of pearl millet was released previously [2]. On the basis of this sequence, pearl millet gene or protein families such as a WRKY transcription factor (TF) family, an NAC (NAM, ATAF and CUC) TF family, a GRAS TF family and a MYB TF family have been identified and characterized [3–6]. However, most pearl millet protein families are uncharacterized. TFs in general regulate transcription of multiple genes and thus can act as hubs for diverse processes. TFs can therefore be useful as either a transgene in genetic modification or a target of genome editing for improving plant performance. The objective of the data presented was to characterize amino acid sequences of most pearl millet TFs.

## Data description

Amino acid sequences for all pearl millet proteins deduced from its whole genome sequence [2] were downloaded from the International Pearl Millet Genome Sequencing Consortium website [7]. Hidden Markov models (HMMs) for protein families in the Pfam database [8] were downloaded from an InterPro website [9]. HMMs in those amino acid sequences were detected by the hmmscan program in HMMER (version 3.3) [10, 11]. On the basis of the detected HMMs, 2395 sequences were regarded as the sequences for putative pearl millet TFs and these were classified into 85 families. Conserved domains in these TFs were confirmed by Batch CD-Search [12, 13]. Subcellular and suborganellar localization of these TFs was predicted by MULocDeep [14, 15]. Amino acid sequences of rice (*Oryza sativa* ssp. *japonica*) and *Arabidopsis thaliana* TFs were downloaded from a PlantTFDB website [16–18]. For the families that were not available in PlantTFDB, amino acid sequences of all rice (*O. sativa* ssp. *indica*) and Arabidopsis proteins were downloaded from an Ensembl Plants website [19, 20] and used for hmmscan as described above to identify proteins in those families. For each of these families except the 13 families which contain less than five members, the sequences from pearl millet, rice and Arabidopsis were aligned by ClustalW [21] and a phylogenetic tree file was obtained with the neighbor-joining method on the MEGA X software [22]. The phylogenetic tree was visualized on the Interactive Tree of Life (iTOL) online tool (version 6) [23, 24]. For each of the 84 families identified, motifs in the pearl millet amino acid sequences were identified *de novo* by the MEME program (version 5.5.0) [25]. Data obtained by these analyses were deposited in the figshare repository (Table 1) [26].

**Table 1:** Overview of data files/data sets

Label	Name of data file/data set	File types (file extension)	Data repository and identifier (DOI or accession number)
Data file 1	seqID_family.txt	Tab-delimited text file (.txt)	figshare (10.6084/m9.figshare.21623829) [26]
Data file 2	family_seqID_w_ol.txt	Tab-delimited text file (.txt)	figshare (10.6084/m9.figshare.21623829) [26]
Data file 3	CD-Search_full_all.txt	Tab-delimited text file (.txt)	figshare (10.6084/m9.figshare.21623829) [26]
Data file 4	CD-Search_wo_domain_define_all.txt	Tab-delimited text file (.txt)	figshare (10.6084/m9.figshare.21623829) [26]
Data file 5	MULocDeep_subcellular_localization_prediction_all.txt	Tab-delimited text file (.txt)	figshare (10.6084/m9.figshare.21623829) [26]
Data file 6	MULocDeep_suborganellar_localization_prediction_all.txt	Tab-delimited text file (.txt)	figshare (10.6084/m9.figshare.21623829) [26]
Data file 7	methods_notes.txt	Text file (.txt)	figshare (10.6084/m9.figshare.21623829) [26]
Data set 1	phylogenetic_trees.zip	Zip archive file (.zip)	figshare (10.6084/m9.figshare.21623829) [26]
Data set 2	MEME_results.zip	Zip archive file (.zip)	figshare (10.6084/m9.figshare.21623829) [26]

## Limitations

- Previous studies on protein family characterization [e.g., 3, 4, 5, 6] were not integrated in the data presented.
- Most protein families other than the TF families in pearl millet are still uncharacterized.

## Data Availability

The data described in this Data note can be freely and openly accessed on figshare under 10.6084/m9.figshare.21623829. Please see Table 1 and references [26] for details and links to the data.

## Abbreviations

TF: Transcription factor
HMM: Hidden Markov model
